# Derivation of mathematical closed form expressions for certain irregular topological indices of 2D nanotubes

**DOI:** 10.1038/s41598-023-38386-1

**Published:** 2023-07-11

**Authors:** Asad Ullah, Shahid Zaman, Arshad Hussain, Asma Jabeen, Melaku Berhe Belay

**Affiliations:** 1 Department of Mathematical Sciences, Karakoram International University Gilgit, Gilgit, 15100 Pakistan; 2 Department of Mathematics, University of Sialkot, Sialkot, 51310 Pakistan; 3 Karakoram International University Gilgit, Hunza Campus, Hunza, Pakistan; 4 Department of Mathematics, University of Management and Technology, Sialkot Campus, Sialkot, Pakistan; 5 Nanotechnology Center of Excellence, Addis Ababa Science and Technology University, P.O.Box 16417, Addis Ababa, Ethiopia

**Keywords:** Computational chemistry, Applied mathematics, Nanoscale materials

## Abstract

A numeric quantity that characterizes the whole structure of a network is called a topological index. In the studies of QSAR and QSPR, the topological indices are utilized to predict the physical features related to the bioactivities and chemical reactivity in certain networks. Materials for 2D nanotubes have extraordinary chemical, mechanical, and physical capabilities. They are extremely thin nanomaterials with excellent chemical functionality and anisotropy. Since, 2D materials have the largest surface area and are the thinnest of all known materials, they are ideal for all applications that call for intense surface interactions on a small scale. In this paper, we derived closed formulae for some important neighborhood based irregular topological indices of the 2D nanotubes. Based on the obtained numerical values, a comparative analysis of these computed indices is also performed.

## Introduction

Carbon nanotubes (CNTs) are actually cylindrical molecules that comprise of rolled-up sheets of single-layer carbon atoms (graphene). They can be single-walled having a less than 1 nm (nm) diameter or multi-walled, comprising of numerous concentrically interlinked nanotubes, with around more than 100 nm diameters. Sumio Iijima discovered the multi-walled carbon nanotubes in 1991^1^. CNTs are bonded with sp^2^ bonds chemically, an extremely strong form of molecular interaction. These nanotubes inherit electrical properties from graphene, which are determined by the rolling-up direction of the graphene layers. Apart from these, CNTs also have distinctive mechanical and thermal properties like light-weight, high tensile strength, low density, better thermal conductivity, high aspect ratio and high chemical stability. All these properties make them intriguing for new materials development, especially CNTs are best candidates for hydrogen storage cells, cathode ray tubes (CRTs), electronic devices, electron field emitters and transistors. Keeping in view their strong applicability and importance, it is very important to model and characterize these CNTs for a better understanding of their structural topology for enhancement of their physical properties.

The study of chemicals using a mathematical method is called mathematical chemistry. Chemical graph theory is a branch of chemistry that uses graph theory concepts to convert chemical events into mathematical models. The chemical graph is a simple connected graph in which atoms and chemical bonds are taken as vertices and edges respectively. A connected graph of order $n = |V(G)|$ and size $m = |E(G)|$ can be created with the help of G and edge set E. The focus of research in the area of nanotechnology is on atoms and Molecules. The Cartesian product of a path graph of m and n is called a 2D lattice.

Graph theory has emerged as a powerful tool for analyzing the structural properties of complex systems represented by graphs. Topological indices, which are numerical quantities derived from graph theory^2–8^, have gained significant attention due to their ability to concisely capture important graph properties. Degree-based topological indices specifically utilize the degrees of vertices in a graph to quantify its structural characteristics^9^.

Degree based indices, such as the Randić index, the atom-bond connectivity index, and the Harary index, capture the connectivity and branching patterns in a graph by considering the distances between pairs of vertices in relation to their degrees^10–14^. These indices have found wide applications in drug design, chemical graph theory, and network analysis^15–18^.

The Zagreb indices, including the first and second Zagreb indices, measure the sum of the vertex degrees and the product of vertex degrees, respectively^19–21^. These degree-based indices have been successfully applied in chemistry, network analysis, and mathematical chemistry. Variants of Zagreb indices, such as the geometric-arithmetic indices and the atom-bond connectivity indices, have been developed to enhance their discriminatory power^22–24^.

Randic-type indices, such as the augmented Zagreb index, the Randic connectivity index, and the atom-bond connectivity indices are derived from degree sequences and capture information regarding vertex degrees^25^. These indices have found applications in chemical graph theory, network analysis, and bioinformatics^26,27^.

Degree-based topological indices have found numerous applications across different disciplines, including chemistry, biology, materials science, and social network analysis. They have been utilized for drug design, chemical property prediction, molecular structure–property relationships, protein classification, community detection, and modeling complex networks^28–30^.

Recent research has focused on developing new degree-based topological indices with enhanced discriminative capabilities and exploring their applications in emerging areas, such as social networks, biological networks, and complex systems. Efforts have also been made to combine degree-based indices with other topological indices to capture more comprehensive structural information. Future directions involve investigating the theoretical properties of degree-based indices, developing efficient algorithms for their computation, and exploring their applications in further real-world problems^31–33^.

The application of Quantity Structure Activity Relationship (QSAR), which links biological structure and activity with certain constraints and properties of molecules as a result, is extensive in biology as well as in the pharmaceutical and medical fields^34,35^. Carbon nanotubes have an intriguing role because of its special application in chemical sciences. The chemical graph theory has found significant role in thousands of topological indicators. The irregularity topological indices are listed in Table 1.

**Table 1:** List of the irregular topological indices.

Introduced by	Notation	Formula
In^36^, Albertson defined the Albertson index (AL)	$AL\left(G\right)$	${\sum }_{uv\epsilon E}\left|{d}_{u}-{d}_{v}\right|$
Vukicevic and Gasparov defined the $IRL$ index in^37^	$IRL\left(\mathrm{G}\right)$	${\sum }_{\mathrm{uv\epsilon E}}|{lnd}_{u}-ln{d}_{v}|$
Abdo et al. defined the total irregularity index (IRRT) in^38^	$IRRT(G)$	$\frac{1}{2}{\sum }_{uv\epsilon E}|{d}_{u}-{d}_{v}|$
Gutman introduced the IRF(G) irregularity index^39^	$IRF\left( G \right)$	$\mathop \sum \limits_{uv\epsilon E} \left( {d_{u} - d_{v} } \right)^{2}$
The Randić index (Li and Gutman)^40^	$\mathrm{IRA}(\mathrm{G})$	$\mathop \sum \limits_{{{\text{uv}}\epsilon{\text{E}}}} \left( {{\text{d}}_{{\text{u}}}^{{\frac{ - 1}{2}}} - {\text{d}}_{{\text{v}}}^{{\frac{ - 1}{2}}} } \right)^{2}$
Reti et al.^41^	$\mathrm{IRDIF}(\mathrm{G})$	${\sum }_{\mathrm{uv\epsilon E}}|\frac{{\mathrm{d}}_{\mathrm{u}}}{{\mathrm{d}}_{\mathrm{v}}}-\frac{{\mathrm{d}}_{\mathrm{v}}}{{\mathrm{d}}_{\mathrm{u}}}|$
	$\mathrm{IRLF}\left(\mathrm{G}\right)$	${\sum }_{\mathrm{uv\epsilon E}}\frac{|{\mathrm{d}}_{\mathrm{u}}-{\mathrm{d}}_{\mathrm{v}}|}{\sqrt{{\mathrm{d}}_{\mathrm{u}}{\mathrm{d}}_{\mathrm{v}}}}$
	$\mathrm{LA}\left(\mathrm{G}\right)$	$2{\sum }_{\mathrm{uv\epsilon E}}\frac{\left|{\mathrm{d}}_{\mathrm{u}}-{\mathrm{d}}_{\mathrm{v}}\right|}{\left({\mathrm{d}}_{\mathrm{u}}+{\mathrm{d}}_{\mathrm{v}}\right)}$
	$\mathrm{IRDI}(\mathrm{G})$	${\sum }_{\mathrm{uv\epsilon E}}\mathrm{ln}\left\{1+|{\mathrm{d}}_{\mathrm{u}}-{\mathrm{d}}_{\mathrm{v}}|\right\}$
Chu and M. Abid have defined the IRGA(G) in^42^	$\mathrm{IRGA}(\mathrm{G})$	${\sum }_{\mathrm{uv\epsilon E}}\mathrm{ln}\frac{{\mathrm{d}}_{\mathrm{u}}+{\mathrm{d}}_{\mathrm{v}}}{2\sqrt{{\mathrm{d}}_{\mathrm{u}}{\mathrm{d}}_{\mathrm{v}}}}$
The bond- additive index is described in^43^	$\mathrm{IRB}(\mathrm{G})$	$\mathop \sum \limits_{{{\text{uv}}\epsilon{\text{E}}}} \left( {{\text{d}}_{{\text{u}}}^{\frac{1}{2}} - {\text{d}}_{{\text{v}}}^{\frac{1}{2}} } \right)^{2}$

Motivated by the above formulas, we have introduced some new neighborhood version of irregular topological indices in Table 2.

**Table 2:** List of the neighborhood version of irregular topological indices.

Notation	Formula
${N}_{AL}\left(G\right)$	${\sum }_{uv\epsilon E}\left|{\delta }_{u}-{\delta }_{v}\right|$
${N}_{IRL}\left(\mathrm{G}\right)$	${\sum }_{\mathrm{uv\epsilon E}}|{ln\delta }_{u}-ln{\delta }_{v}|$
${N}_{IRRL}\left(G\right)$	$\frac{1}{2}{\sum }_{uv\epsilon E}\left|{\delta }_{u}-{\delta }_{v}\right|$
${N}_{IRF}(G)$	$\mathop \sum \limits_{uv\epsilon E} \left( {\delta_{u} - \delta_{v} } \right)^{2}$
${N}_{IRA}(\mathrm{G})$	$\mathop \sum \limits_{{{\text{uv}}\epsilon{\text{E}}}} \left( {\delta_{{\text{u}}}^{{\frac{ - 1}{2}}} - \delta_{{\text{v}}}^{{\frac{ - 1}{2}}} } \right)^{2}$
${N}_{IRDIF}(\mathrm{G})$	${\sum }_{\mathrm{uv\epsilon E}}|\frac{{\delta }_{\mathrm{u}}}{{\delta }_{\mathrm{v}}}-\frac{{\delta }_{\mathrm{v}}}{{\delta }_{\mathrm{u}}}|$
${N}_{IRLF}\left(\mathrm{G}\right)$	${\sum }_{\mathrm{uv\epsilon E}}\frac{|{\delta }_{\mathrm{u}}-{\delta }_{\mathrm{v}}|}{\sqrt{{\delta }_{\mathrm{u}}{\delta }_{\mathrm{v}}}}$
${N}_{LA}(\mathrm{G})$	$2{\sum }_{\mathrm{uv\epsilon E}}\frac{\left|{\delta }_{\mathrm{u}}-{\delta }_{\mathrm{v}}\right|}{\left({\delta }_{\mathrm{u}}+{\delta }_{\mathrm{v}}\right)}$
${N}_{IRDI}\mathrm{G})$	${\sum }_{\mathrm{uv\epsilon E}}\mathrm{ln}\left\{1+|{\delta }_{\mathrm{u}}-{\delta }_{\mathrm{v}}|\right\}$
${N}_{IRGA}(\mathrm{G})$	${\sum }_{\mathrm{uv\epsilon E}}\mathrm{ln}\frac{{\delta }_{\mathrm{u}}+{\delta }_{\mathrm{v}}}{2\sqrt{{\delta }_{\mathrm{u}}{\delta }_{\mathrm{v}}}}$
${N}_{IRB}(\mathrm{G})$	$\mathop \sum \limits_{{{\text{uv}}\epsilon{\text{E}}}} \left( {\delta_{{\text{u}}}^{\frac{1}{2}} - \delta_{{\text{v}}}^{\frac{1}{2}} } \right)^{2}$

Numerous efforts have been made to investigate the topological indices for various nanotubes and nanosheets in the literature. The topological invariants of Pent-Heptagonal nanosheets and TURC_4_C_8_(S) are studied respectively in^44,45^. The topological indices of V-phenylenic type nanotori and nanotubes have been discussed in^46^, and armchair polyhex type nanotube in^47^. For detailed insights into the investigations on topological modeling and analysis of micro and nanostructures, one might consult refs^27,30,32,48–62^. Despite all these investigations, the Nano structural topology has not yet been unveiled completely. In this study, we derived closed formulae for some neighborhood version of irregular topological indices of the nanotubes $HA{C}_{5}{C}_{7}[p, q]$ and $HA{C}_{5}{\mathrm{C}}_{6}{\mathrm{C}}_{7}[\mathrm{p},\mathrm{q}]$, and performed a comparative analysis based on the numerical results.

## The HAC_5_C_7_[p, q] nanotubes (p, q > 1)

**Figure 1:**
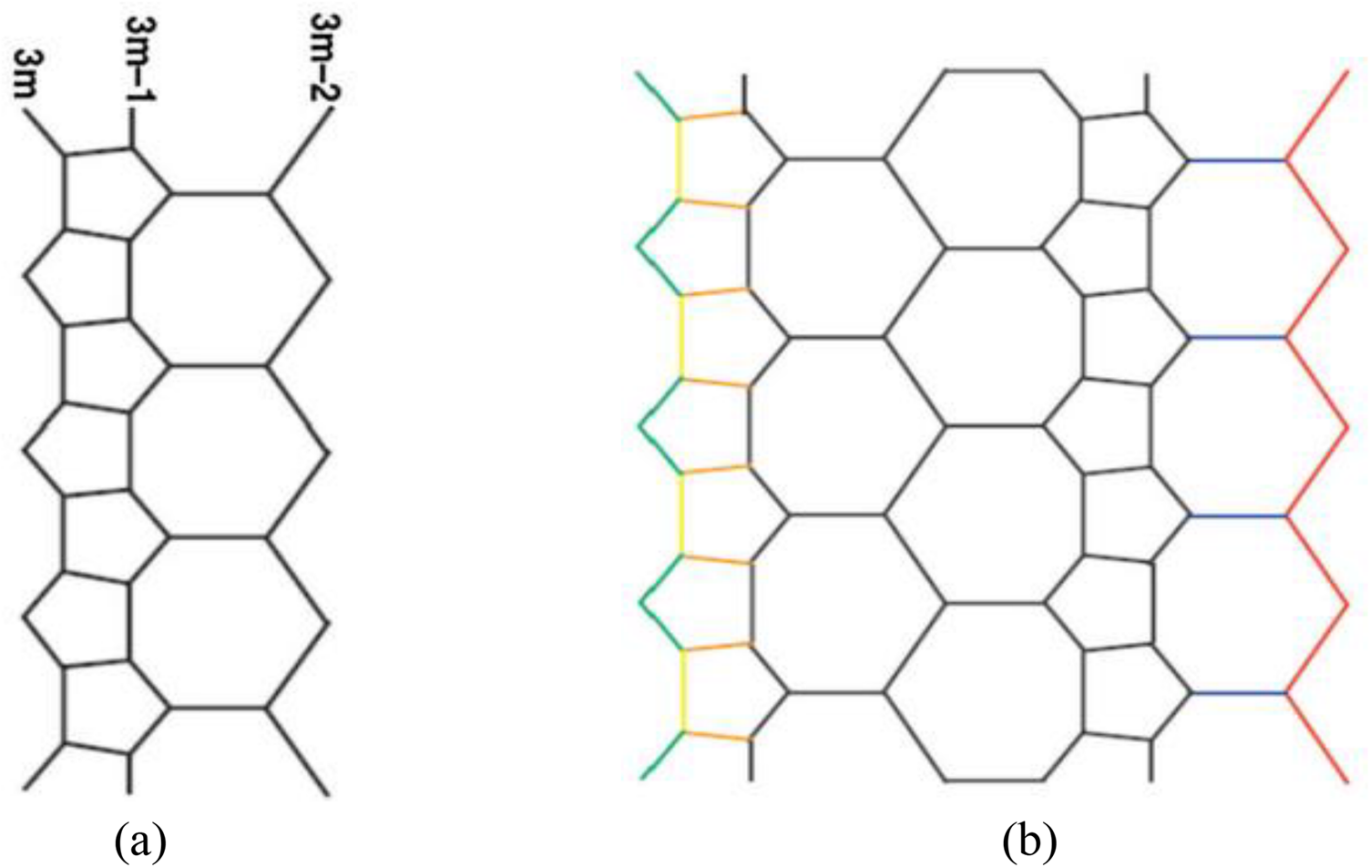
(**a**) The HAC_5_C_7_ nanotube –mth period. (**b**) The HAC_5_C_7_ nanotube with p = 4, q = 2.

A trivalent adornment has remained complete by joining ${C}_{5}$ and ${C}_{7}$ and recognized as ${C}_{5}{C}_{7}$ net. It has been utilized to conceal both a tube and a torus. As a ${C}_{5}{C}_{7}$ net, the $HA{C}_{5}{C}_{7}[p, q]$ nanotube can be studied. In 2007, Iranmanesh and Khormali calculated the vertex–Szeged index of $HA{C}_{5}{C}_{7}$ nanotube. The two dimensional lattice of $HA{C}_{5}{C}_{7}$ has been explained consistently. In the entire lattice, the number of heptagons and period are represented by p and q in row. There are $8pq+p$ vertices and $12pq-p$ edges, respectively . The three rows of $HA{C}_{5}{C}_{7}$ is said to be m^th^ period﻿ (Fig. 1). Consider the graph of $HA{C}_{5}{C}_{7}$ is represented by G . The cardinality of vertex set is $8pq+p$ and edge set is $12pq-p$ for the graph G. The vertex set is divided into three categories based on their degrees. The order of vertex ${V}_{1}$ is 8pq. Similarly, $|{V}_{2}|=2p+2$ ,$\left|{V}_{3}\right|=8pq-p-2$. In the whole study, we denote the adjacent vertices by p and q, i.e. $pq\in {E}_{G}.$ The edge set is divided into the subsequent sections according to their sum of neighborhood degree, called the frequency, which is shown in Table 3.

**Table 3:** The neighborhood edge partitions of $HA{C}_{5}{C}_{7}[p ,q]$.

$({\updelta }_{\mathrm{p}} ,{\updelta }_{\mathrm{q}})$	Frequency
$(\mathrm{9,9})$	$12\mathrm{pq}-9\mathrm{p}$
$(\mathrm{9,8})$	$2\mathrm{p}$
$(\mathrm{9,7})$	$\mathrm{p}$
$(\mathrm{8,8})$	$\mathrm{p}$
$(\mathrm{8,6})$	$2\mathrm{p}$
$(\mathrm{7,6})$	$2\mathrm{p}$

### Theorem 1

Assume that $G\in {\mathrm{HAC}}_{5}{\mathrm{C}}_{7}[\mathrm{p },\mathrm{q}]$ then, ${N}_{AL}(\mathrm{G})= 10\mathrm{p}$

### Proof

By definition of ${N}_{AL}\left(\mathrm{G}\right)$ and from the neighborhood edge partitions in Table 3, one has

$$N_{AL} \left( {\text{G}} \right) = \mathop \sum \limits_{{{\text{pq}}\epsilon{\text{E}}}} \left| {{\updelta }_{{\text{p}}} - {\updelta }_{{\text{q}}} } \right|$$

$$= \left( {12{\text{pq}} - 9{\text{p}}} \right)\left| {9 - 9} \right| + \left( {2{\text{p}}} \right)\left| {9 - 8\left| { + {\text{p}}} \right|9 - 7\left| { + {\text{p}}} \right|8 - 8} \right| + \left( {2{\text{p}}} \right)\left| {8 - 6} \right| + \left( {2{\text{p}}} \right)\left| {7 - 6} \right|$$

$$= 2{\text{p}}\left( 1 \right) + {\text{p}}\left( 2 \right) + 2{\text{p}}\left( 2 \right) + 2{\text{p}}\left( 1 \right)$$

$$= 2{\text{p}} + 2{\text{p}} + 4{\text{p}} + 2{\text{p}}$$

$$N_{AL} \left( {\text{G}} \right) = { }10{\text{p}}$$

### Theorem 2

Assume that $G\in {\mathrm{HAC}}_{5}{\mathrm{C}}_{7}[\mathrm{p },\mathrm{q}]$ then, ${N}_{IRL}(\mathrm{G})=1.3705867\mathrm{p}$

### Proof

Similar to the proof of theorem 1, one has

$$N_{IRL} \left( G \right) = \mathop \sum \limits_{pq\epsilon E} |\ln \delta_{p} - \ln \delta_{q} |$$

$$= \left( {12{\text{pq}} - 9{\text{p}}} \right)\left| {{\text{ln}}9 - {\text{ln}}9} \right| + 2{\text{p}}\left| {{\text{ln}}9 - {\text{ln}}8\left| { + {\text{p}}} \right|{\text{ln}}9 - {\text{ln}}7\left| { + {\text{p}}} \right|{\text{ln}}8 - {\text{ln}}8} \right| + 2{\text{p}}\left| {{\text{ln}}8 - {\text{ln}}6} \right| + 2{\text{p}}\left| {{\text{ln}}7 - {\text{ln}}6} \right|$$

$$= 2{\text{p}}\left( {2.197224 - 2.079441} \right) + {\text{p}}\left( {2.19724 - 1.94591} \right) + 2{\text{p}}\left( {2.07944 - 1.79175} \right) + 2{\text{p}}\left( {1.945910 - 1.79175} \right)$$

$$= 2{\text{p}}\left( {0.1177835} \right) + {\text{p}}\left( {0.25133} \right) + 2{\text{p}}\left( {0.28769} \right) + 2{\text{p}}\left( {0.15416} \right)$$

$$= 0.235567{\text{p}} + 0.57538{\text{p}} + 0.30832{\text{p}} + 0.25133{\text{p}}$$

$$N_{IRL} \left( {\text{G}} \right) = 1.3705867{\text{p}}$$

### Theorem 3

Assume that $G\in {\mathrm{HAC}}_{5}{\mathrm{C}}_{7}[\mathrm{p },\mathrm{q}]$ then, ${N}_{IRRT}(G)=5p$

### Proof

Based on Table 3 and the definition of ${N}_{IRRT}$ we have

$$N_{IRRT} \left( {\text{G}} \right) = \frac{1}{2}\mathop \sum \limits_{{{\text{pq}}\epsilon{\text{E}}}} \left| {{\updelta }_{{\text{p}}} - {\updelta }_{{\text{q}}} } \right|$$

$$= \left( {12{\text{pq}} - 9{\text{p}}} \right)\frac{1}{2}\left| {9 - 9} \right| + 2{\text{p}}\frac{1}{2}\left| {9 - 8} \right| + {\text{p}}\frac{1}{2}\left| {9 - 7} \right| + {\text{p}}\frac{1}{2}\left| {8 - 8} \right| + 2{\text{p}}\frac{1}{2}\left| {8 - 6} \right| + 2{\text{p}}\frac{1}{2}\left| {7 - 6} \right|$$

$$= {\text{p}} + {\text{p}} + 2{\text{p}} + {\text{p}}$$

$$N_{IRRT} \left( {\text{G}} \right) = 5{\text{p}}$$

### Theorem 4

Assume that $G\in {\mathrm{HAC}}_{5}{\mathrm{C}}_{7}[\mathrm{p },\mathrm{q}]$ then, ${N}_{IRF}(\mathrm{G})=16\mathrm{p}$

### Proof

Together Table 3 with the definition $N_{IRF} \left( {\text{G}} \right) = \mathop \sum \limits_{{{\text{pq}}\epsilon{\text{E}}}} \left( {{\updelta }_{{\text{p}}} - {\updelta }_{q} } \right)^{2}$, one has

$$= \left( {12{\text{pq}} - 9{\text{p}}} \right)\left( {9 - 9} \right)^{2} + 2{\text{p}}\left( {9 - 8} \right)^{2} + {\text{p}}\left( {9 - 7} \right)^{2} + {\text{p}}\left( {8 - 8} \right)^{2} + 2{\text{p}}\left( {8 - 6} \right)^{2} + 2{\text{p}}\left( {7 - 6} \right)^{2}$$

$$= 2{\text{p}} + 4{\text{p}} + 2{\text{p}}\left( 4 \right) + 2{\text{p}}$$

$$= 2{\text{p}} + 4{\text{p}} + 8{\text{p}} + 2{\text{p}}$$

$$N_{IRF} \left( {\text{G}} \right) = 16{\text{p}}$$

### Theorem 5

Assume that $G\in {\mathrm{HAC}}_{5}{\mathrm{C}}_{7}[\mathrm{p },\mathrm{q}]$ then, ${N}_{IRA}(\mathrm{G})=0.0106268538\mathrm{p}$

### Proof

By definition $$N_{IRA} \left( {\text{G}} \right) = \mathop \sum \limits_{{{\text{pq}}\epsilon{\text{E}}}} \left( {{\updelta }_{{\text{p}}}^{{\frac{ - 1}{2}}} - {\updelta }_{{\text{q}}}^{{\frac{ - 1}{2}}} } \right)^{2}$$

$$= \left( {12{\text{pq}} - 9{\text{p}}} \right)\left( {9^{{\frac{ - 1}{2}}} - 9^{{\frac{ - 1}{2}}} } \right)^{2} + 2{\text{p}}\left( {9^{{\frac{ - 1}{2}}} - 8^{{\frac{ - 1}{2}}} } \right)^{2} + {\text{p}}\left( {9^{{\frac{ - 1}{2}}} - 7^{{\frac{ - 1}{2}}} } \right)^{2} + {\text{p}}\left( {8^{{\frac{ - 1}{2}}} - 8^{{\frac{ - 1}{2}}} } \right)^{2} + 2{\text{p}}\left( {8^{{\frac{ - 1}{2}}} - 6^{{\frac{ - 1}{2}}} } \right)^{2} + 2{\text{p}}\left( {7^{{\frac{ - 1}{2}}} - 6^{{\frac{ - 1}{2}}} } \right)^{2}$$

$$= 2{\text{p}}\left( {0.333333 - 0.353553} \right)^{2} + {\text{p}}\left( {0.33333 - 0.37796} \right)^{2} + 2{\text{p}}\left( {0.353553 - 0.408248} \right)^{2} + 2{\text{p}}\left( {0.377964 - 0.408248} \right)^{2}$$

$$= 0.0008176968{\text{p}} + 0.001991836{\text{p}} + 0.00598308{\text{p}} + 0.001834241{\text{p}}$$

$${\text{IRA}}\left( {\text{G}} \right) = 0.0106268538{\text{p}}$$

### Theorem 6

Assume that $G\in {\mathrm{HAC}}_{5}{\mathrm{C}}_{7}[\mathrm{p },\mathrm{q}]$ then, ${N}_{IRDIF}(\mathrm{G})=2.765846\mathrm{p}$

### Proof

By definition $${N}_{IRDIF}(\mathrm{G})={\sum }_{\mathrm{pq\epsilon E}}|\frac{{\updelta }_{\mathrm{p}}}{{\updelta }_{\mathrm{q}}}-\frac{{\updelta }_{\mathrm{q}}}{{\updelta }_{\mathrm{p}}}|$$

$$= \left( {12{\text{pq}} - 9{\text{p}}} \right)\left| {\frac{9}{9} - \frac{9}{9}\left| { + 2{\text{p}}} \right|\frac{9}{8} - \frac{8}{9}\left| { + {\text{p}}} \right|\frac{9}{7} - \frac{7}{9}\left| { + {\text{p}}} \right|\frac{8}{8} - \frac{8}{8}\left| { + 2{\text{p}}} \right|\frac{8}{6} - \frac{6}{8}\left| { + 2{\text{p}}} \right|\frac{7}{6} - \frac{6}{7}} \right|$$

$$= 2{\text{p}}\left( {1.125 - 0.88889} \right) + {\text{p}}\left( {1.28571 - 0.77778} \right) + 2{\text{p}}\left( {1.33333 - 0.75} \right) + 2{\text{p}}\left( {1.166667 - 0.857142} \right)$$

$$= 0.47222{\text{p}} + 0.50791{\text{p}} + 1.1666667{\text{p}} + 0.619056{\text{p}}$$

$$N_{IRDIF} \left( {\text{G}} \right) = 2.765846{\text{p}}$$

### Theorem 7

Assume that $G\in {\mathrm{HAC}}_{5}{\mathrm{C}}_{7}[\mathrm{p },\mathrm{q}]$ then, ${N}_{IRLF}(\mathrm{G})=1.37363426\mathrm{p}$

### Proof

By definition $${N}_{IRLF}(\mathrm{G})={\sum }_{\mathrm{uv\epsilon E}}\frac{|{\updelta }_{\mathrm{p}}-{\updelta }_{\mathrm{q}}|}{\sqrt{{\updelta }_{\mathrm{p}}{\updelta }_{q}}}$$

$$= \left( {12{\text{pq}} - 9{\text{p}}} \right)\frac{{\left| {9 - 9} \right|}}{{\sqrt {9 \times 9} }} + \left( {2{\text{p}}} \right)\frac{{\left| {9 - 8} \right|}}{{\sqrt {9 \times 8} }} + {\text{p}}\frac{{\left| {9 - 7} \right|}}{{\sqrt {9 \times 7} }} + {\text{p}}\frac{{\left| {8 - 8} \right|}}{{\sqrt {8 \times 8} }} + 2{\text{p}}\frac{{\left| {8 - 6} \right|}}{{\sqrt {8 \times 6} }} + 2{\text{p}}\frac{{\left| {7 - 6} \right|}}{{\sqrt {7 \times { }6} }}$$

$$= 2{\text{p}}\frac{1}{{\sqrt {72} }} + {\text{p}}\frac{2}{{\sqrt {63} }} + 2{\text{p}}\frac{2}{{\sqrt {48} }} + 2{\text{p}}\frac{1}{{\sqrt {42} }}$$

$$= {\text{p}}\left( {0.23570226} \right) + \left( {0.251976} \right) + {\text{p}}\left( {0.57735} \right) + {\text{p}}\left( {0.308606} \right)$$

$$N_{IRLF} \left( {\text{G}} \right) = 1.37363426{\text{p}}$$

### Theorem 8

Assume that $G\in {\mathrm{HAC}}_{5}{\mathrm{C}}_{7}[\mathrm{p },\mathrm{q}]$ then, ${N}_{LA}(\mathrm{G})=1.36441441\mathrm{p}$

### Proof

By definition $${N}_{LA}(\mathrm{G})={\sum }_{\mathrm{pq\epsilon E}}\frac{|{\updelta }_{\mathrm{p}}-{\updelta }_{\mathrm{q}}|}{({\updelta }_{\mathrm{p}}+{\updelta }_{\mathrm{q}})}$$

$$= \left( {12{\text{pq}} - 9{\text{p}}} \right)2\frac{{\left| {9 - 9} \right|}}{{\left( {9 + 9} \right)}} + \left( {2{\text{p}}} \right)2\frac{{\left| {9 - 8} \right|}}{{\left( {9 + 8} \right)}} + \left( {\text{p}} \right)2\frac{{\left| {9 - 7} \right|}}{{\left( {9 + 7} \right)}} + \left( {\text{p}} \right)2\frac{{\left| {8 - 8} \right|}}{{\left( {8 + 8} \right)}} + 2{\text{p}}\left( 2 \right)\frac{{\left| {8 - 6} \right|}}{{\left( {8 + 6} \right)}} + 2{\text{p}}\left( 2 \right)\frac{{\left| {7 - 6} \right|}}{{\left( {7 + 6} \right)}}$$

$$= 4{\text{p}}\frac{1}{17} + 2{\text{p}}\frac{2}{16} + 4{\text{p}}\frac{2}{14} + 4{\text{p}}\frac{1}{13}$$

$$= 0.23529411{\text{p}} + 0.25{\text{p}} + 0.571428{\text{p}} + 0.30769230{\text{p}}$$

$$N_{LA} \left( {\text{G}} \right) = 1.36441441{\text{p}}$$

### Theorem 9

Assume that $G\in {\mathrm{HAC}}_{5}{\mathrm{C}}_{7}[\mathrm{p },\mathrm{q}]$ then, ${N}_{IRDI}(\mathrm{G})=6.068425221\mathrm{p}$

### Proof

By definition $${N}_{IRDI}(\mathrm{G})= {\sum }_{\mathrm{pq\epsilon E}}\mathrm{ln}(1+\left|{\updelta }_{\mathrm{p}}-{\updelta }_{\mathrm{q}}\right|)$$

$$= \left( {12{\text{pq}} - 9{\text{p}}} \right){\text{ln}}\left( {1 + \left| {9 - 9} \right|} \right) + 2{\text{pln}}\left( {1 + \left| {9 - 8} \right|} \right){\text{pln}}\left( {1 + \left| {9 - 7} \right|} \right) + {\text{pln}}\left( {1 + \left| {8 - 8} \right|} \right) + 2{\text{pln}}\left( {1 + \left| {8 - 6} \right|} \right) + 2{\text{pln}}\left( {1 + \left| {7 - 6} \right|} \right)$$

$$= \left( {12{\text{pq}} - 9{\text{p}}} \right){\text{ln}}1 + 2{\text{pln}}2 + {\text{pln}}3 + {\text{pln}}1 + 2{\text{pln}}3 + 2{\text{pln}}2$$

$$= 1.38629436{\text{p}} + 1.098612{\text{p}} + 2.19972245{\text{p}} + 1.38629436{\text{p}}$$

$$N_{IRDI} \left( {\text{G}} \right) = 6.068425221{\text{p}}$$

### Theorem 10

Assume that $G\in {\mathrm{HAC}}_{5}{\mathrm{C}}_{7}[\mathrm{p },\mathrm{q}]$ then, ${N}_{IRGA}\left(\mathrm{G}\right)=1.26918503\mathrm{p}$

### Proof

By definition $${N}_{IRGA}\left(\mathrm{G}\right)={\sum }_{\mathrm{uv\epsilon E}}\frac{\mathrm{ln}|{\updelta }_{p}+{\updelta }_{\mathrm{q}}|}{2\sqrt{{\updelta }_{\mathrm{p}}{\updelta }_{\mathrm{q}}}}$$

$$= \left( {12{\text{pq}} - 9{\text{p}}} \right){\text{ln}}\frac{{\left| {9 + 9} \right|}}{{2\sqrt {9 \times 9} }} + 2{\text{pln}}\frac{{\left| {9 + 8} \right|}}{{2\sqrt {9 \times 8} }} + {\text{pln}}\frac{{\left| {9 + 7} \right|}}{{2\sqrt {9 \times 7} }} + {\text{pln}}\frac{{\left| {8 + 8} \right|}}{{2\sqrt {8 \times 8} }} + 2{\text{pln}}\frac{{\left| {8 + 6} \right|}}{{2\sqrt {8 \times 6} }} + 2{\text{pln}}\frac{{\left| {7 + 6} \right|}}{{2\sqrt {7 \times 6} }}$$

$$= 2{\text{p}}\left( {0.066766} \right) + 2{\text{p}}\left( {0.696112778} \right) + 2{\text{p}}\left( {0.001733104307} \right) + {\text{p}}\left( {0.0070252649} \right)$$

$$= 0.0034662086{\text{p}} + 0.0070252649{\text{p}} - 0.133532{\text{p}} + 1.392225556{\text{p}}$$

$$N_{IRGA} \left( {\text{G}} \right) = 1.26918503{\text{p}}$$

### Theorem 11

Assume that $G\in {\mathrm{HAC}}_{5}{\mathrm{C}}_{7}[\mathrm{p },\mathrm{q}]$ then, ${N}_{IRB} (\mathrm{G})=0.5486855\mathrm{p}$

### Proof

By definition $$N_{IRB} { }\left( {\text{G}} \right) = \mathop \sum \limits_{{{\text{pq}}\epsilon{\text{E}}}} \left( {{\updelta }_{p}^{\frac{1}{2}} - {\updelta }_{q}^{\frac{1}{2}} } \right)^{2}$$

$$= \left( {12{\text{pq}} - 9{\text{p}}} \right)\left( {9^{\frac{1}{2}} - 9^{\frac{1}{2}} } \right)^{2} + 2{\text{p}}\left( {9^{\frac{1}{2}} - 8^{\frac{1}{2}} } \right)^{2} + {\text{p}}\left( {9^{\frac{1}{2}} - 7^{\frac{1}{2}} } \right)^{2} + {\text{p}}\left( {8^{\frac{1}{2}} - 8^{\frac{1}{2}} } \right)^{2} + 2{\text{p}}\left( {8^{\frac{1}{2}} - 6^{\frac{1}{2}} } \right)^{2} + 2{\text{p}}\left( {7^{\frac{1}{2}} - 6^{\frac{1}{2}} } \right)^{2}$$

$$= 0.058874{\text{p}} + 0.125492{\text{p}} + 0.287282{\text{p}} + 0.0770375{\text{p}}$$

$$N_{IRB} { }\left( {\text{G}} \right) = 0.5486855{\text{p}}$$

## The HAC_5_C_6_C_7_[p, q] nanotubes (p, q > 1)

Let G be the graph of ${\mathrm{HAC}}_{5}{\mathrm{C}}_{6}{\mathrm{C}}_{7}\left[\mathrm{p},\mathrm{ q}\right]$ nanotube. Then,

### Theorem 12

**Figure 2:**
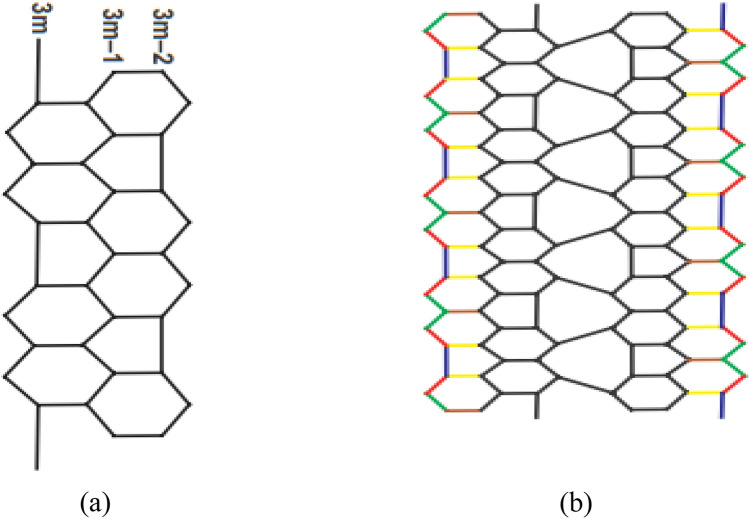
(**a**) The HAC_5_C_6_C_7_ nanotube mth period, (**b**) The HAC_5_C_6_C_7_ nanotube with p = 4 and q = 2.

Assume that $G\in {\mathrm{HAC}}_{5}{\mathrm{C}}_{6}{\mathrm{C}}_{7}\left[\mathrm{p},\mathrm{ q}\right]$ be a graph as shown in Fig. 2. Then, ${N}_{AL}(\mathrm{G})=18\mathrm{p}$

### Proof

By definition of ${N}_{AL}(\mathrm{G})$ and Table 4 one has:

$$N_{AL} \left( {\text{G}} \right) = \mathop \sum \limits_{{{\text{pq}}\epsilon{\text{E}}}} \left| {{\updelta }_{{\text{p}}} - {\updelta }_{{\text{q}}} } \right|$$

$$= \left( {12{\text{pq}} - 9{\text{p}}} \right)\left| {9 - 9} \right| + 4{\text{p}}\left| {9 - 8} \right| + 2{\text{p}}\left| {8 - 8} \right| + 2{\text{p}}\left| {8 - 7} \right| + 4{\text{p}}\left| {8 - 6} \right| + 4{\text{p}}\left| {7 - 6} \right|$$

$$= 4{\text{p}} + 2{\text{p}} + 8{\text{p}} + 4{\text{p}}$$

$$N_{AL} \left( {\text{G}} \right) = 18{\text{p}}$$

**Table 4:** The neighborhood edge partitions of HAC_5_C_6_C_7_ nanotube.

$({\updelta }_{p}, {\updelta }_{q}$)	Frequency
$(\mathrm{9,9})$	$12pq-9p$
$(\mathrm{9,8})$	$4p$
$(\mathrm{8,8})$	$2p$
$(\mathrm{8,7})$	$2p$
$(\mathrm{8,6})$	$4p$
$(\mathrm{7,6})$	$4p$

### Theorem 13

Assume that $G\in {\mathrm{HAC}}_{5}{\mathrm{C}}_{6}{\mathrm{C}}_{7}\left[\mathrm{p},\mathrm{ q}\right]$ be a graph as shown in Fig. 2. Then, ${N}_{IRL}\left(\mathrm{G}\right)=9\mathrm{p}$

### Proof

By definition $${N}_{IRL}\left(\mathrm{G}\right)={\sum }_{\mathrm{pq}\in \mathrm{E}}|\mathrm{ln}{\updelta }_{\mathrm{p}}-\mathrm{ln}{\updelta }_{\mathrm{q}}|$$

$$= \left( {12{\text{pq}} - 9{\text{p}}} \right)\left| {{\text{ln}}9 - {\text{ln}}9} \right| + 4{\text{p}}\left| {{\text{ln}}9 - {\text{ln}}8} \right| + 2{\text{p}}\left| {{\text{ln}}8 - {\text{ln}}8} \right| + 2{\text{p}}\left| {{\text{ln}}8 - {\text{ln}}7} \right| + 4{\text{p}}\left| {{\text{ln}}8 - {\text{ln}}6} \right| + 4{\text{p}}\left| {{\text{ln}}7 - {\text{ln}}6} \right|$$

$$= 4{\text{p}}\left( {2.197224577734 - 2.07944154168} \right) + 2{\text{p}}\left( {2.0794415 - 1.94591014906} \right) + 4{\text{p}}\left( {1.94591014906 - 1.79175946} \right)$$

$$= 0.47113214264{\text{p}} + 0.26706270188{\text{p}} + 1.15072832672{\text{p}} + 0.61660275624{\text{p}}$$

$$N_{IRL} \left( {\text{G}} \right) = 2.505525927{\text{p}}$$

### Theorem 14

Assume that $G\in {\mathrm{HAC}}_{5}{\mathrm{C}}_{6}{\mathrm{C}}_{7}\left[\mathrm{p},\mathrm{ q}\right]$ be a graph as shown in Fig. 2. Then, ${N}_{IRRT}(\mathrm{G})=9\mathrm{p}$

### Proof

By definition $${N}_{IRRT}(G)=\frac{1}{2}{\sum }_{pq\epsilon E}\left|{\updelta }_{p}-{\updelta }_{q}\right|$$

$$= 4{\text{p}}\frac{1}{2}\left| {9 - 8} \right| + 2{\text{p}}\frac{1}{2}\left| {8 - 8} \right| + 2{\text{p}}\frac{1}{2}\left| {8 - 7} \right| + 4{\text{p}}\frac{1}{2}\left| {8 - 6} \right| + 4{\text{p}}\frac{1}{2}\left| {7 - 6} \right|$$

$$= 2{\text{p}} + {\text{p}} + 4{\text{p}} + 2{\text{p}}$$

$$N_{IRRT} \left( {\text{G}} \right) = 9{\text{p}}$$

### Theorem 15

Assume that $G\in {\mathrm{HAC}}_{5}{\mathrm{C}}_{6}{\mathrm{C}}_{7}\left[\mathrm{p},\mathrm{ q}\right]$ be a graph as shown in Fig. 2. Then, ${N}_{IRF}(\mathrm{G})=26\mathrm{p}$

### Proof

By definition $$N_{IRF} \left( {\text{G}} \right) = \mathop \sum \limits_{{{\text{pq}}\epsilon{\text{E}}}} \left( {{\updelta }_{{\text{p}}} - {\updelta }_{{\text{q}}} } \right)^{2}$$

$$= 4{\text{p}}\left( {9 - 8} \right)^{2} + 2{\text{p}}\left( {8 - 8} \right)^{2} + 2{\text{p}}\left( {8 - 7} \right)^{2} + 4{\text{p}}\left( {8 - 6} \right)^{2} + 4{\text{p}}\left( {7 - 6} \right)^{2}$$

$$= 4{\text{p}} + 2{\text{p}} + 16{\text{p }} + 4{\text{p}}$$

$$N_{IRF} \left( {\text{G}} \right) = 26{\text{p}}$$

### Theorem 16

Assume that $G\in {\mathrm{HAC}}_{5}{\mathrm{C}}_{6}{\mathrm{C}}_{7}\left[\mathrm{p},\mathrm{ q}\right]$ be a graph as shown in Fig. 2. Then, ${N}_{IRA}\left(\mathrm{G}\right)=0.0184628432\mathrm{p}$

### Proof

By definition $$N_{IRA} \left( {\text{G}} \right) = \mathop \sum \limits_{{{\text{pq}}\epsilon{\text{E}}}} \left( {{\updelta }_{{\text{p}}}^{{\frac{ - 1}{2}}} - {\updelta }_{{\text{q}}}^{{\frac{ - 1}{2}}} } \right)^{2}$$

$$= \left( {12{\text{pq}} - 9{\text{p}}} \right)\left( {9^{{\frac{ - 1}{2}}} - 9^{{\frac{ - 1}{2}}} } \right)^{2} + 4{\text{p}}\left( {9^{{\frac{ - 1}{2}}} - 8^{{\frac{ - 1}{2}}} } \right)^{2} + 2{\text{p}}\left( {8^{{\frac{ - 1}{2}}} - 8^{{\frac{ - 1}{2}}} } \right)^{2} + 2{\text{p}}\left( {8^{{\frac{ - 1}{2}}} - 7^{{\frac{ - 1}{2}}} } \right)^{2} + 4{\text{p}}\left( {8^{{\frac{ - 1}{2}}} - 6^{{\frac{ - 1}{2}}} } \right)^{2} + 4{\text{p}}\left( {7^{{\frac{ - 1}{2}}} - 6^{{\frac{ - 1}{2}}} } \right)^{2}$$

$$= 4{\text{p}}\left( {0.33333 - 0.353553} \right)^{2} + 2{\text{p}}\left( {0.353553 - 0.37796} \right)^{2} + 4{\text{p}}\left( {0.353553 - 0.408248} \right)^{2} + 4{\text{p}}\left( {0.37796 - 0.408248} \right)^{2}$$

$$= 4{\text{p}}\left( {0.00040896} \right) + 2{\text{p}}\left( {0.0005957016} \right) + 4{\text{p}}\left( {0.00299154} \right) + 4{\text{p}}\left( {0.00091736} \right)$$

$$= 0.00163584{\text{p}} + 0.0011914032{\text{p}} + 0.01196616{\text{p}} + 0.00366944{\text{p}}$$

$$N_{IRA} \left( {\text{G}} \right) = 0.0184628432{\text{p}}$$

### Theorem 17

Assume that $G\in {\mathrm{HAC}}_{5}{\mathrm{C}}_{6}{\mathrm{C}}_{7}\left[\mathrm{p},\mathrm{ q}\right]$ be a graph as shown in Fig. 2. Then, ${N}_{IRDIF}\mathrm{G})=5.051528\mathrm{p}$

### Proof

By definition $${N}_{IRDIF}(\mathrm{G})={\sum }_{\mathrm{pq\epsilon E}}|\frac{{\updelta }_{\mathrm{p}}}{{\updelta }_{\mathrm{q}}}-\frac{{\updelta }_{\mathrm{q}}}{{\updelta }_{\mathrm{p}}}|$$

$$= \left( {12{\text{pq}} - 9{\text{p}}} \right)\left| {\frac{9}{9} - \frac{9}{9}} \right| + 4{\text{p}}\left| {\frac{9}{8} - \frac{8}{9}} \right| + 2{\text{p}}\left| {\frac{8}{8} - \frac{8}{8}} \right| + 2{\text{p}}\left| {\frac{8}{7} - \frac{7}{8}} \right| + 4{\text{p}}\left| {\frac{8}{6} - \frac{6}{8}} \right| + 4{\text{p}}\left| {\frac{7}{6} - \frac{6}{7}} \right|$$

$$= 4{\text{p}}\left( {1.125 - 0.888889} \right) + 2{\text{p}}\left( {1.14285 - 0.875} \right) + 4{\text{p}}\left( {1.3333 - 0.75} \right) + 4{\text{p}}\left( {1.1666667 - 0.85714} \right)$$

$$= 0.9444{\text{p}} + 0.5357{\text{p}} + 2.333332{\text{p}} + 1.238108{\text{p}}$$

$$N_{IRDIF} \left( {\text{G}} \right) = 5.051528{\text{p}}$$

### Theorem 18

Assume that $G\in {\mathrm{HAC}}_{5}{\mathrm{C}}_{6}{\mathrm{C}}_{7}\left[\mathrm{p},\mathrm{ q}\right]$ be a graph as shown in Fig. 2. Then, ${N}_{IRLF}(\mathrm{G})=2.510484\mathrm{p}$

### Proof

By definition $${N}_{IRLF}(\mathrm{G})={\sum }_{\mathrm{pq\epsilon E}}\frac{|{\updelta }_{\mathrm{p}}-{\updelta }_{\mathrm{q}}|}{\sqrt{{\updelta }_{\mathrm{p}}{\updelta }_{q}}}$$

$$= \left( {12{\text{pq}} - 9{\text{p}}} \right)\frac{{\left| {9 - 9} \right|}}{{\sqrt {9 \times 9} }} + 4{\text{p}}\frac{{\left| {9 - 8} \right|}}{{\sqrt {9 \times 8} }} + 2{\text{p}}\frac{{\left| {8 - 8} \right|}}{{\sqrt {8 \times 8} }} + 2{\text{p}}\frac{{\left| {8 - 7} \right|}}{{\sqrt {8 \times 7} }} + 4{\text{p}}\frac{{\left| {8 - 6} \right|}}{{\sqrt {8 \times 6} }} + 4{\text{p}}\frac{{\left| {7 - 6} \right|}}{{\sqrt {7 \times 6} }}$$

$$= 4{\text{p}}\left( {0.117851} \right) + 2{\text{p}}\left( {0.1336} \right) + 4{\text{p}}\left( {0.28867} \right) + 4{\text{p}}\left( {0.15430} \right)$$

$$= 0.471404{\text{p}} + 0.2672{\text{p}} + 1.15468{\text{p}} + 0.6172{\text{p}}$$

$$N_{IRLF} \left( {\text{G}} \right) = 2.510484{\text{p}}$$

### Theorem 19

Assume that $G\in {\mathrm{HAC}}_{5}{\mathrm{C}}_{6}{\mathrm{C}}_{7}\left[\mathrm{p},\mathrm{ q}\right]$ be a graph as shown in Fig. 2. Then, ${N}_{LA}(\mathrm{G})=2.495499845\mathrm{p}$

### Proof

By definition $${N}_{LA}(\mathrm{G})={\sum }_{\mathrm{pq\epsilon E}}\frac{|{\updelta }_{\mathrm{p}}-{\updelta }_{\mathrm{q}}|}{({\updelta }_{\mathrm{p}}+{\updelta }_{\mathrm{q}})}$$

$$= \left( {12{\text{pq}} - 9{\text{p}}} \right)2\frac{{\left| {9 - 9} \right|}}{{\left( {9 + 9} \right)}} + 4{\text{p}}\left( 2 \right)\frac{{\left| {9 - 8} \right|}}{{\left( {9 + 8} \right)}} + 2{\text{p}}\left( 2 \right)\frac{{\left| {8 - 8} \right|}}{{\left( {8 + 8} \right)}} + 2{\text{p}}\left( 2 \right)\frac{{\left| {8 - 7} \right|}}{{\left( {8 + 7} \right)}} + 4{\text{p}}\left( 2 \right)\frac{{\left| {8 - 6} \right|}}{{\left( {8 + 6} \right)}} + 4{\text{p}}\left( 2 \right)\frac{{\left| {7 - 6} \right|}}{{\left( {7 + 6} \right)}}$$

$$= 8{\text{p}}\frac{1}{17} + 4{\text{p}}\frac{1}{15} + 8{\text{p}}\frac{2}{14} + 8{\text{p}}\frac{1}{13}$$

$$= 0.47058823{\text{p}} + 0.266667{\text{p}} + 1.142857{\text{p}} + 0.615384{\text{p}}$$

$$N_{LA} \left( {\text{G}} \right) = 2.495499845{\text{p}}$$

### Theorem 20

Assume that $G\in {\mathrm{HAC}}_{5}{\mathrm{C}}_{6}{\mathrm{C}}_{7}\left[\mathrm{p},\mathrm{ q}\right]$ be a graph as shown in Fig. 2. Then, ${N}_{IRDI}(\mathrm{G})=8.553331516\mathrm{p}$

### Proof

By definition $${N}_{IRDI}(\mathrm{G})= {\sum }_{\mathrm{pq\epsilon E}}\mathrm{ln}(1+\left|{\updelta }_{\mathrm{p}}-{\updelta }_{\mathrm{q}}\right|)$$

$$= \left( {12{\text{pq}} - 9{\text{p}}} \right){\text{ln}}\left( {1 + \left| {9 - 9} \right|} \right) + 4{\text{pln}}\left( {1 + \left| {9 - 8} \right|} \right) + 2{\text{pln}}\left( {1 + \left| {8 - 8} \right|} \right) + 2{\text{pln}}(1 + \left| {8 - 7} \right| + 4{\text{pln}}\left( {1 + \left| {8 - 6} \right|} \right) + 4{\text{pln}}\left( {1 + \left| {7 - 6} \right|} \right)$$

$$= 4{\text{pln}}\left( {1 + 1} \right) + 2{\text{pln}}\left( {1 + 1} \right) + 4{\text{pln}}\left( {1 + 2} \right) + 4{\text{pln}}\left( {1 + 1} \right)$$

$$= 4{\text{pln}}2 + 2{\text{pln}}2 + 4{\text{pln}}3 + 4{\text{pln}}2$$

$$= 2.772588{\text{p}} + 1.386294361{\text{p}} + 4.3944915{\text{p}}4$$

$$N_{IRDI} \left( {\text{G}} \right) = 8.553331516{\text{p}}$$

### Theorem 21

Assume that $G\in {\mathrm{HAC}}_{5}{\mathrm{C}}_{6}{\mathrm{C}}_{7}\left[\mathrm{p},\mathrm{ q}\right]$ be a graph as shown in Fig. 2. Then, ${N}_{IRGA}(\mathrm{G})=0.06449422104\mathrm{p}$

### Proof

By definition $${N}_{IRGA}(\mathrm{G})={\sum }_{\mathrm{pq\epsilon E}}\frac{\mathrm{ln}|{\updelta }_{\mathrm{p}}+{\updelta }_{\mathrm{q}}|}{2\sqrt{{\updelta }_{\mathrm{p}}{\updelta }_{\mathrm{q}}}}$$

$$= \left( {12{\text{pq}} - 9{\text{p}}} \right){\text{ln}}\frac{{\left| {9 + 9} \right|}}{{2\sqrt {9 \times 9} }} + 4{\text{pln}}\frac{{\left| {9 + 8} \right|}}{{2\sqrt {9 \times 8} }} + 2{\text{pln}}\frac{{\left| {8 + 8} \right|}}{{2\sqrt {8 \times 8} }} + 2{\text{pln}}\frac{{\left| {8 + 7} \right|}}{{2\sqrt {8 \times 7} }} + 4{\text{pln}}\frac{{\left| {8 + 6} \right|}}{{2\sqrt {8 \times 6} }} + 4{\text{pln}}\frac{{\left| {7 + 6} \right|}}{{2\sqrt {7 \times 6} }}$$

$$= 4{\text{pln}}\frac{17}{{2\sqrt {72} }} + 2{\text{pln}}\frac{15}{{2\sqrt {50} }} + 4{\text{pln}}\frac{14}{{2\sqrt {48} }} + 4{\text{pln}}\frac{13}{{2\sqrt {42} }}$$

$$= 0.0069324{\text{p}} + 0.00445435{\text{p}} + 0.041238{\text{p}} + 0.01186947{\text{p}}$$

$$N_{IRGA} \left( {\text{G}} \right) = 0.06449422104{\text{p}}$$

### Theorem 22

Assume that $G\in {\mathrm{HAC}}_{5}{\mathrm{C}}_{6}{\mathrm{C}}_{7}\left[\mathrm{p},\mathrm{ q}\right]$ be a graph as shown in Fig. 2. Then, $N_{IRB} \left( G \right) = 0.9130008{\text{p}}$

### Proof

By definition $$N_{IRB} \left( {\text{G}} \right) = \mathop \sum \limits_{{{\text{pq}}\epsilon{\text{E}}}} \left( {{\updelta }_{{\text{p}}}^{\frac{1}{2}} - {\updelta }_{{\text{q}}}^{\frac{1}{2}} } \right)^{2}$$

$$= \left( {12{\text{pq}} - 9{\text{p}}} \right)\left( {9^{\frac{1}{2}} - 9^{\frac{1}{2}} } \right)^{2} + 4{\text{p}}\left( {9^{\frac{1}{2}} - 8^{\frac{1}{2}} } \right)^{2} + 2{\text{p}}\left( {8^{\frac{1}{2}} - 8^{\frac{1}{2}} } \right)^{2} + 2{\text{p}}\left( {8^{\frac{1}{2}} - 7^{\frac{1}{2}} } \right)^{2} + 4{\text{p}}\left( {8^{\frac{1}{2}} - 6^{\frac{1}{2}} } \right)^{2} + 4{\text{p}}\left( {7^{\frac{1}{2}} - 6^{\frac{1}{2}} } \right)^{2}$$

$$= 4{\text{p}}\left( {3 - 2.828427} \right)^{2} + 2{\text{p}}\left( {2.828427 - 2.645751} \right)^{2} + 4{\text{p}}\left( {2.828427 - 2.449489} \right)^{2} + 4{\text{p}}\left( {2.6457 - 2.4494} \right)^{2}$$

$$= 0.117749{\text{p}} + 0.06674104{\text{p}} + 0.574376{\text{p}} + 0.15413476{\text{p}}$$

$${N}_{IRB}(\mathrm{G})=0.9130008\mathrm{p}$$

### Numerical discussion and conclusion

**Figure 3:**
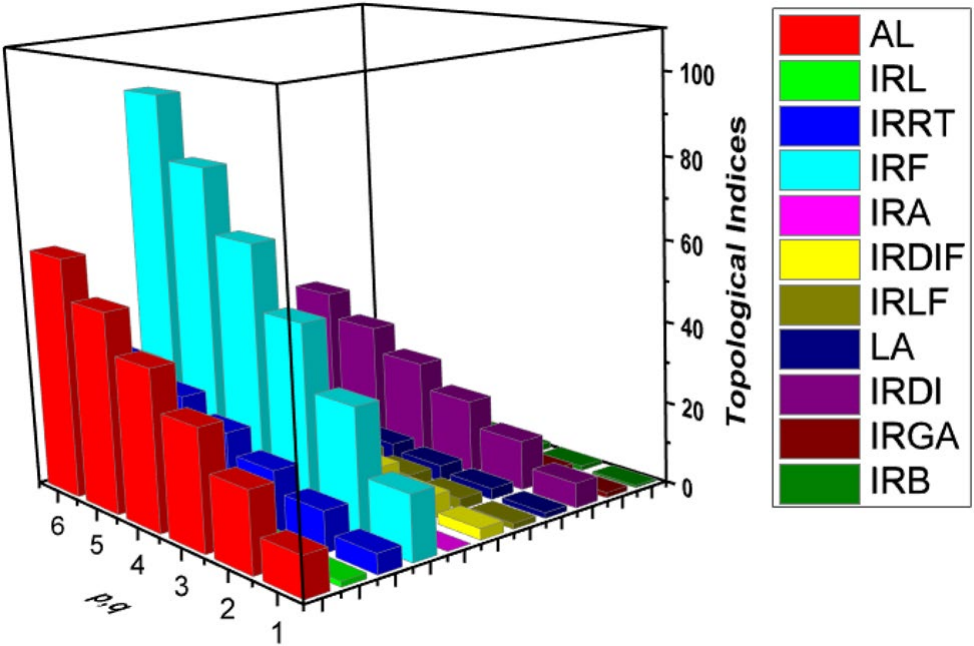
Comparison graph for $HA{C}_{5}{\mathrm{C}}_{7}[\mathrm{p},\mathrm{q}]$ nanotube.

In this section, we conclude our work with some important remarks. In Section "The HAC_5_C_7_[p, q] nanotubes (p, q > 1)" we constructed the structures of HAC5C7[p, q] nanotubes for $p,q>1$. Based on Fig. 1a, b, we obtained the neighborhood edge partitions as shown in Table 3. With the help of these partitions, we determined the neighborhood irregularity topological indices. Moreover, the numerical and graphical comparisons among all considered topological indices are given in Table 5 and Fig. 3. Which shows that there is a positive relation between p, q and these topological indices. That is to say, when we increase the values of p and q the values of topological indices also increase. Hence, from this comparison it is easy to see that the value of ${N}_{IRF}$ index is higher than the values of remaining topological indices.

**Table 5:** Comparison of computed indices for $HA{C}_{5}{\mathrm{C}}_{7}[\mathrm{p},\mathrm{q}]$ nanotube.

$\left[ {{\varvec{p}},{\varvec{q}}} \right]$	${\varvec{N}}_{{{\varvec{AL}}}}$	${\varvec{N}}_{{{\varvec{IRL}}}}$	${\varvec{N}}_{{{\varvec{IRRT}}}}$	${\varvec{N}}_{{{\varvec{IRF}}}}$	${\varvec{N}}_{{{\varvec{IRA}}}}$	${\varvec{N}}_{{{\varvec{IRDIF}}}}$	${\varvec{N}}_{{{\varvec{IRLF}}}}$	${\varvec{N}}_{{{\varvec{LA}}}}$	${\varvec{N}}_{{{\varvec{IRDI}}}}$	${\varvec{N}}_{{{\varvec{IRGA}}}}$	${\varvec{N}}_{{{\varvec{IRB}}}}$
$\left[ {1,1} \right]$	$10$	$1.37$	$5$	$16$	$0.01$	$2.76$	$1.37$	$1.36$	$6.06$	$1.26$	$0.54$
$\left[ {2,2} \right]$	$20$	$2.74$	$10$	$32$	$0.02$	$5.53$	$2.75$	$2.72$	$12.13$	$2.53$	$1.09$
$\left[ {3,3} \right]$	$30$	$4.11$	$15$	$48$	$0.03$	$8.29$	$4.12$	$4.09$	$18.20$	$3.80$	$1.64$
$\left[ {4,4} \right]$	$40$	$5.48$	$20$	$64$	$0.04$	$11.06$	$5.54$	$5.45$	$24.27$	$5.07$	$2.19$
$\left[ {5,5} \right]$	$50$	$6.87$	$25$	$80$	$0.05$	$13.82$	$6.86$	$6.82$	$30.34$	$6.34$	$2.74$
$\left[ {6,6} \right]$	$60$	$8.25$	$30$	$96$	$0.06$	$16.59$	$8.24$	$8.18$	$36.41$	$7.61$	$3.2$

**Figure 4:**
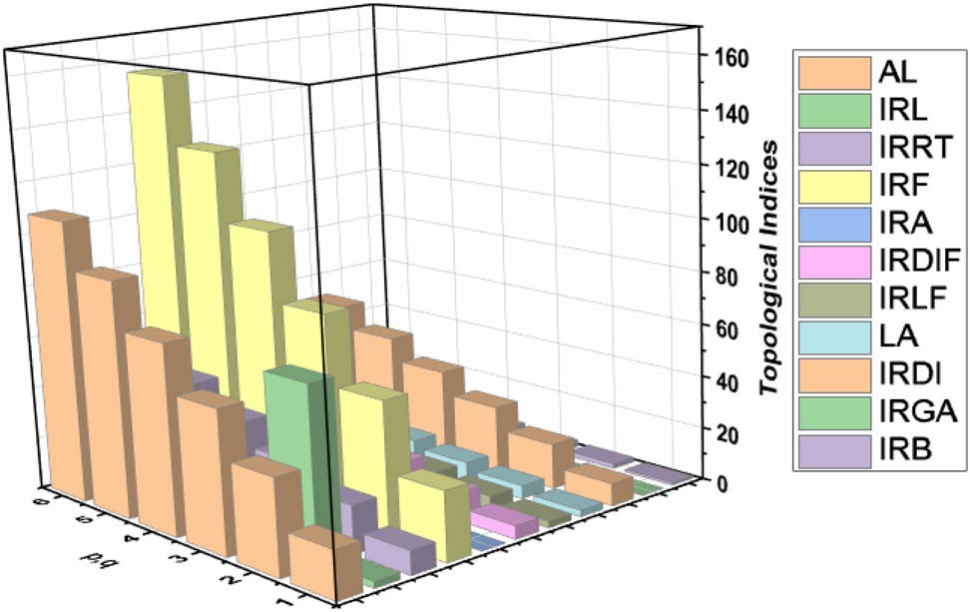
Comparison graph for $HA{C}_{5}{\mathrm{C}}_{6}{\mathrm{C}}_{7}[\mathrm{p},\mathrm{q}]$ nanotube.

In Section "The HAC_5_C_6_C_7_[p, q] nanotubes (p, q > 1)", we constructed the structures of ${\mathrm{HAC}}_{5}{\mathrm{C}}_{6}{\mathrm{C}}_{7}\left[\mathrm{p},\mathrm{ q}\right]$ nanotubes for p,q > 1. Based on Fig. 2a, b, we obtained the edge partitions as shown in Table 4. With the help of these edge partitions, we determined the neighborhood irregularity topological indices. Moreover, the numerical and graphical comparisons among all considered topological indices are given in Table 6 and Fig. 4. Which shows that there is a positive relation between p, q and these topological indices, when we increase the values of p and q, the values of topological indices also increase. Hence, from this comparison it is easy to see that the value of ${N}_{IRF}$ index is higher than the values of remaining topological indices.

**Table 6:** Comparison of computed indices for $HA{C}_{5}{\mathrm{C}}_{6}{\mathrm{C}}_{7}[\mathrm{p},\mathrm{q}]$ nanotube.

$[p,q]$	${N}_{AL}$	${N}_{IRL}$	${N}_{IRRT}$	${N}_{IRF}$	${N}_{IRA}$	${N}_{IRDIF}$	${N}_{IRLF}$	${N}_{LA}$	${N}_{IRDI}$	${N}_{IRGA}$	${N}_{IRB}$
$[\mathrm{1,1}]$	$18$	$2.50$	$9$	$26$	$0.01$	$5.05$	$2.51$	$2.49$	$8.55$	$0.06$	$0.91$
$[\mathrm{2,2}]$	$36$	$65.04$	$18$	$52$	$0.03$	$10.10$	$5.020$	$4.99$	$17.10$	$0.12$	$1.82$
$[\mathrm{3,3}]$	$54$	$7.51$	$27$	$78$	$0.05$	$15.15$	$7.53$	$7.48$	$25.65$	$0.19$	$2.73$
$[\mathrm{4,4}]$	$72$	$10.02$	$36$	$104$	$0.07$	$20.20$	$10.04$	$9.98$	$34.21$	$0.25$	$3.65$
$[\mathrm{5,5}]$	$90$	$12.52$	$45$	$130$	$0.09$	$25.25$	$12.55$	$12.47$	$42.76$	$0.32$	$4.56$
$[\mathrm{6,6}]$	$108$	$15.03$	$54$	$156$	$0.12$	$30.30$	$15.06$	$14.97$	$51.31$	$0.38$	$5.47$

## Data Availability

All data generated or analysed during this study are included in this article.
